# Thromboembolic risk and factor Xa inhibitor use in nephrotic syndrome: a pilot study in a predominantly Hispanic cohort

**DOI:** 10.1093/ckj/sfag274

**Published:** 2026-08-12

**Authors:** Palacios-Cuervo Fernando, Andre Crestani, Debnath Subrata, Carlos Lorenzo, Tareq Nassar, Swetha Rani Kanduri

**Affiliations:** Division of Nephrology, Department of Medicine, University of Texas Health Science Center at San Antonio, San Antonio, TX, USA; Division of Nephrology, Department of Medicine, University of Texas Health Science Center at San Antonio, San Antonio, TX, USA; Division of Nephrology, Department of Medicine, University of Texas Health Science Center at San Antonio, San Antonio, TX, USA; Division of Rheumatology, Department of Medicine, University of Texas Health Science Center at San Antonio, San Antonio, TX, USA; Division of Nephrology, Department of Medicine, University of Texas Health Science Center at San Antonio, San Antonio, TX, USA; Division of Nephrology, Department of Medicine, University of Texas Health Science Center at San Antonio, San Antonio, TX, USA

To the Editor,

Venous thromboembolism (VTE) is a serious complication of nephrotic syndrome (NS), with risk influenced by serum albumin, proteinuria, and the underlying glomerular disease subtype [1]. Membranous nephropathy (MN) has traditionally been considered the glomerular disease subtype with the highest thrombotic risk. Current KDIGO guidelines recommend consideration of prophylactic anticoagulation in selected high-risk patients. However, these recommendations are largely based on studies of MN and have limited validation across other histopathologic subtypes [2]. Furthermore, Hispanic patients have been underrepresented in prior studies and the evidence regarding direct oral anticoagulants, particularly factor Xa inhibitors, in NS remains limited [3].

We conducted a pilot study to characterize VTE incidence, clinical risk factors, and anticoagulation outcomes in adults with biopsy-confirmed NS in a predominantly Hispanic population. We retrospectively reviewed adults with biopsy-confirmed glomerulonephritis-associated NS treated at UT Health San Antonio from 2015 to 2025 with ≥12 months of follow-up. Patients with major confounders for VTE were excluded. The primary outcome was imaging-confirmed VTE. VTE incidence was evaluated by baseline serum albumin, glomerular disease subtype, and anticoagulation strategy using standard statistical tests in SAS software.

Of 70 patients screened, 29 met inclusion criteria. The mean age was 47.7 years, 58.6% were male, and 55.2% were Hispanic. Hypertension was present in 51.7%. Mean baseline serum albumin was 1.33 g/dl, and 65.5% had severe hypoalbuminemia (≤1.3 g/dl). Five patients (17.2%) developed objectively confirmed VTE during follow-up. Patients with VTE were older than those without VTE (63.4 vs. 44.5 years; *P *= 0.030) and had greater kidney dysfunction, including higher serum creatinine (3.18 vs. 1.13 mg/dl; *P *= 0.002) and lower estimated glomerular filtration rate (30.8 vs. 82.4 ml/min/1.73 m²; *P *= 0.016). All VTE events occurred in patients with serum albumin ≤1.3 g/dl. VTE incidence was 26.3% (5/19) among patients with albumin ≤1.3 g/dl compared with 0% (0/10) among those with albumin >1.3 g/dl (likelihood-ratio *P *= 0.029) (Table S1 in the Supplementary file).

VTE incidence differed by histologic subtype. The highest event rate occurred in amyloidosis (66.7%), followed by minimal change disease (28.6%) and lupus nephritis (14.3%). No VTE events occurred among patients with MN or focal segmental glomerulosclerosis.

Among 17 patients who received no anticoagulation, 3 (17.6%) developed VTE. Two of five patients (40.0%) receiving prophylactic heparin/enoxaparin developed VTE, whereas none of seven patients receiving apixaban developed VTE (Fig. 1). No major bleeding events were observed among patients receiving apixaban.

**Figure 1:**
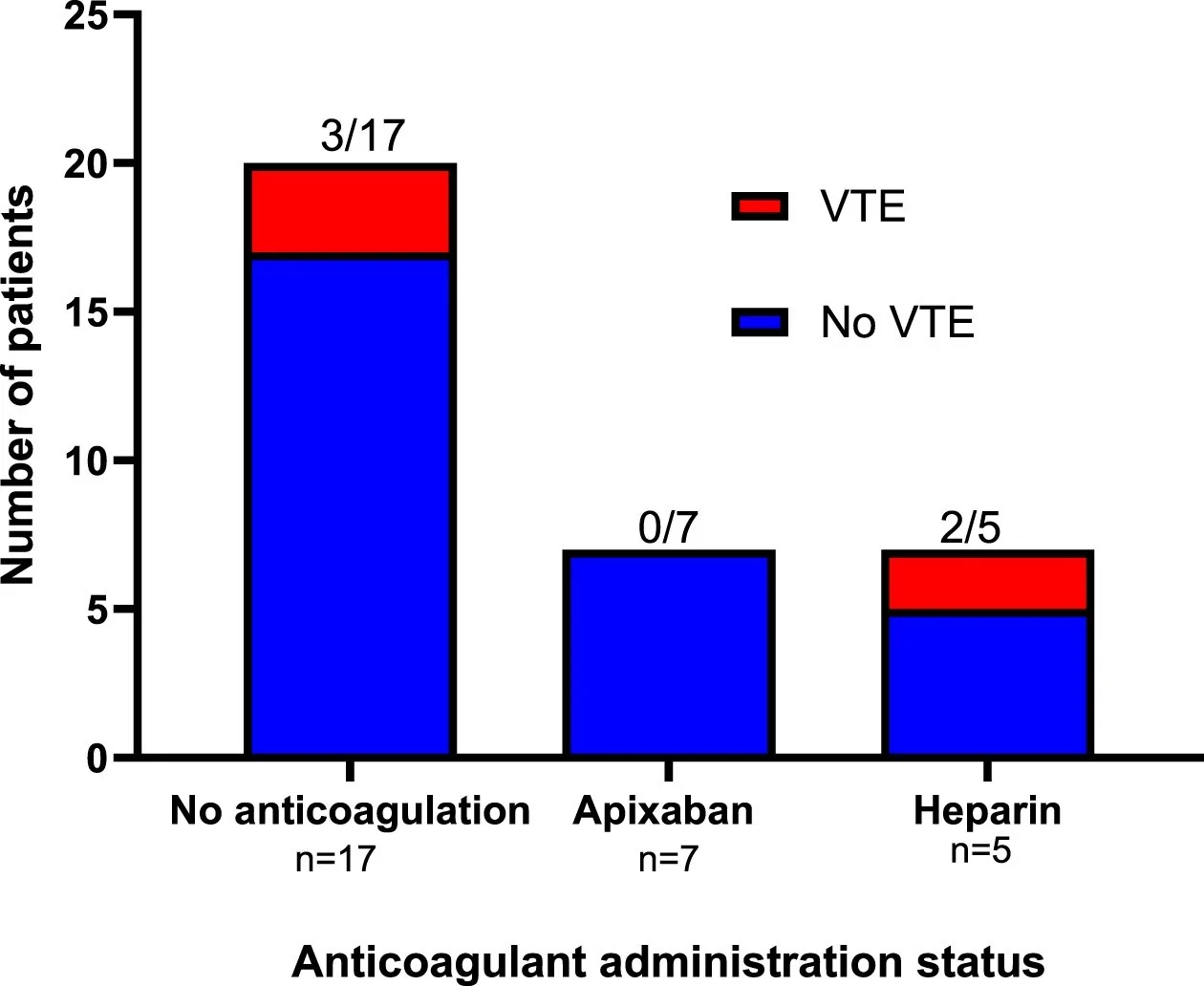
Frequency of VTE episodes based on anticoagulation. VTE occurred in 3 of 17 patients (17.6%) who did not receive anticoagulation and in 2 of 5 patients (40.0%) who received prophylactic heparin/enoxaparin. No VTE events were observed among the seven patients treated with apixaban (0%).

Despite KDIGO recommendations based on low-quality evidence with favorable benefit–risk ratios at low albumin levels, prophylactic anticoagulation has been inconsistently applied in real-world practice. In this pilot cohort, severe hypoalbuminemia was strongly associated with thromboembolic risk, with all VTE events occurring at serum albumin levels ≤1.3 g/dl. Older age and impaired kidney function were associated with VTE, suggesting that affected patients may represent a clinically higher-risk phenotype.

A unique aspect of this cohort was the substantial representation of Hispanic patients, a population underrepresented in prior NS studies. While MN has traditionally been considered the highest-risk glomerular disease subtype for VTE, no thromboembolic events occurred among patients with MN in this cohort [4]. Instead, the highest event rates were observed among patients with amyloidosis and minimal change disease (MCD). The reasons for this unexpected distribution remain uncertain and may relate to differences in disease severity, biological characteristics, genetic factors, or other unmeasured clinical variables within Hispanic population. However, larger studies are needed to validate these results.

Similar to some recent studies [5], no VTE events occurred among patients receiving apixaban prophylaxis in the current study and no major bleeding events were observed. Larger prospective studies are needed to validate albumin-based risk thresholds, evaluate safety and efficacy of apixaban, and determine whether ethnicity influences thrombotic risk across glomerular disease subtypes.

## Supplementary Material

sfag274_Supplemental_File
