# Supplementary material for: Thromboembolic risk and factor Xa inhibitor use in nephrotic syndrome: a pilot study in a predominantly Hispanic cohort
Source: Clin Kidney J. 2026 Aug 12;19(9):sfag274. doi: 10.1093/ckj/sfag274 (PMC13569861; doi:10.1093/ckj/sfag274)
Supplement: Supplementary file 1 [file sfag274_supplemental_file.docx]

| Variable | Overall Cohort (n=29) | No VTE (n=24) | VTE (n=5) |
| --- | --- | --- | --- |
| Age, years, mean (range) | 47.7 (21–79) | 44.5 (21–75) | 63.4 (41–79) |
| Male sex, n (%) | 17 (58.6%) | 13 (54.2%) | 4 (80.0%) |
| Hispanic ethnicity, n (%) | 16 (55.2%) | — | — |
| Hypertension, n (%) | 15 (51.7%) | — | — |
| Albumin baseline, g/dL, mean | 1.33 | 1.39 | 1.06 |
| Albumin ≤1.3 g/dL, n (%) | 19 (65.5%) | 14 (58.3%) | 5 (100%) |
| Serum Creatinine, mg/dL, mean | 1.48 | 1.13 | 3.18* |
| BUN, mg/dL, mean | 24.6 | 21.4 | 40.0* |
| eGFR, mean | 73.4 | 82.4 | 30.8* |
| Hemoglobin, g/dL, mean | 11.8 | 12.3 | 9.0 |

**Table 1.** Baseline patient characteristics overall and stratified by VTE status. * p<0.05 VTE vs. No VTE group (Wilcoxon two-sample test).

**p* < 0.05 vs. No VTE group (Wilcoxon two-sample test).

**Abbreviations:** VTE, venous thromboembolism; BUN, blood urea nitrogen; eGFR, estimated glomerular filtration rate; g/dL, grams per deciliter; mg/dL, milligrams per deciliter; and n, number of patients.
